# HIV-1 Protease in the Fission Yeast *Schizosaccharomyces pombe*

**DOI:** 10.1371/journal.pone.0151286

**Published:** 2016-03-16

**Authors:** Zsigmond Benko, Robert T. Elder, Ge Li, Dong Liang, Richard Y. Zhao

**Affiliations:** 1 Department of Pathology, University of Maryland Medical School, Baltimore, Maryland, United States of America; 2 Department of Microbiology-Immunology, University of Maryland Medical School, Baltimore, Maryland, United States of America; 3 Institute of Human Virology, University of Maryland Medical School, Baltimore, Maryland, United States of America; 4 Chicago Memorial Institute for Education and Research, Northwestern University Feinberg School of Medicine, Chicago, Illinois, United States of America; New York University College of Dentistry, UNITED STATES

## Abstract

**Background:**

HIV-1 protease (PR) is an essential viral enzyme. Its primary function is to proteolyze the viral Gag-Pol polyprotein for production of viral enzymes and structural proteins and for maturation of infectious viral particles. Increasing evidence suggests that PR cleaves host cellular proteins. However, the nature of PR-host cellular protein interactions is elusive. This study aimed to develop a fission yeast (*Schizosaccharomyces pombe*) model system and to examine the possible interaction of HIV-1 PR with cellular proteins and its potential impact on cell proliferation and viability.

**Results:**

A fission yeast strain RE294 was created that carried a single integrated copy of the *PR* gene in its chromosome. The *PR* gene was expressed using an inducible *nmt1* promoter so that PR-specific effects could be measured. HIV-1 PR from this system cleaved the same indigenous viral p6/MA protein substrate as it does in natural HIV-1 infections. HIV-1 *PR* expression in fission yeast cells prevented cell proliferation and induced cellular oxidative stress and changes in mitochondrial morphology that led to cell death. Both these PR activities can be prevented by a PR-specific enzymatic inhibitor, indinavir, suggesting that PR-mediated proteolytic activities and cytotoxic effects resulted from enzymatic activities of HIV-1 PR. Through genome-wide screening, a serine/threonine kinase, Hhp2, was identified that suppresses HIV-1 PR-induced protease cleavage and cell death in fission yeast and in mammalian cells, where it prevented PR-induced apoptosis and cleavage of caspase-3 and caspase-8.

**Conclusions:**

This is the first report to show that HIV-1 protease is functional as an enzyme in fission yeast, and that it behaves in a similar manner as it does in HIV-1 infection. HIV-1 PR-induced cell death in fission yeast could potentially be used as an endpoint for mechanistic studies, and this system could be used for developing a high-throughput system for drug screenings.

## Introduction

HIV-1 protease (PR) is an aspartic protease that is normally present as a homodimer with individual subunits of 99 amino acids (12 kD) [1]. The active enzymatic site lies between the identical subunits and can be blocked by specific and competitive PR inhibitors (PI) such as indinavir (IDV) [2, 3]. The primary function of HIV-1 PR is to proteolyze the viral Gag-Pol polyprotein for the production of viral enzymes (reverse transcriptase, PR, and integrase) and structural proteins, as well as for the maturation of infectious viral particles [4–7]. Thus, PR is an essential viral enzyme that contributes to viral reproduction. Because of the important role of HIV-1 PR in HIV-1 infection, it is a major therapeutic target for antiretroviral therapies (ARTs). Indeed, HIV-1 PI is currently the most potent class of anti-HIV drugs. Monotherapy with PI alone can reduce HIV-1 viral loads by several logs [8]. When a PI drug is used in combination with other classes of anti-HIV drugs to treat HIV-infected patients, HIV-1 viral loads could be constrained to the lowest possible level, which often cannot be detected by conventional laboratory methods.

Besides proteolysis of HIV-1 viral proteins, PR also cleaves host cellular targets, including various cellular kinases [9–12], suggesting intimate interactions between HIV-1 PR and host cellular proteins. In fact, among all of the HIV-1 proteins, PR has been associated with the greatest number of host factors [9]. Although the molecular mechanism and virologic relevance of these interactions are not fully understood at the moment, HIV-1 PR apparently induces necrotic and apoptotic cell death in CD4+ T cells and other cell types, indicating that it may, at least in part, contribute to CD4+ T cell depletion [13, 14]. There is also an intriguing possibility that interactions between HIV-1 PR and host cellular proteins might represent another viral strategy to evade host cellular and/or immune defenses [12]. Thus, the goal of this study was to examine the possible interactions of HIV-1 PR with cellular proteins and the impacts of these interactions on cell proliferation and viability.

Fission yeast (*Schizosaccharomyces pombe*) was chosen for the initial study for several reasons. Fission yeast is a simple and unicellular organism with cellular functions that in many ways resemble mammalian cells [15, 16]. For example, fission yeast has been used broadly as a model system to study cell cycle regulation, chromosomal biology, and cell death/apoptosis, and knowledge gained from this model organism has contributed significantly to the field of cancer biology [17–20]. Fission yeast has also been used as a model system in our laboratory and in others to study the effects of HIV-1 viral protein R (Vpr) on cell cycle G2/M regulation, nuclear transport, and cell death/apoptosis. Many new aspects of this viral protein have been revealed that would otherwise be difficult to discover simply by studying mammalian cells [21–30]. In one of our recent efforts to systematically characterize viral proteins of the entire HIV-1 genome in fission yeast, HIV-1 PR exhibited a significant effect on yeast cell proliferation [31]. Since the effect of HIV-1 PR on fission yeast functions has not been reported previously, the objective of this study was to study the effect of HIV-1 PR on cell proliferation and survival as well as possible cellular protein-PR interactions in fission yeast. Potential implications of the new findings learned from fission yeast on mammalian cells were also explored.

## Results

### Expression of HIV-1 *PR* prevents cell proliferation and colony formation in fission yeast

**Fig 1:**
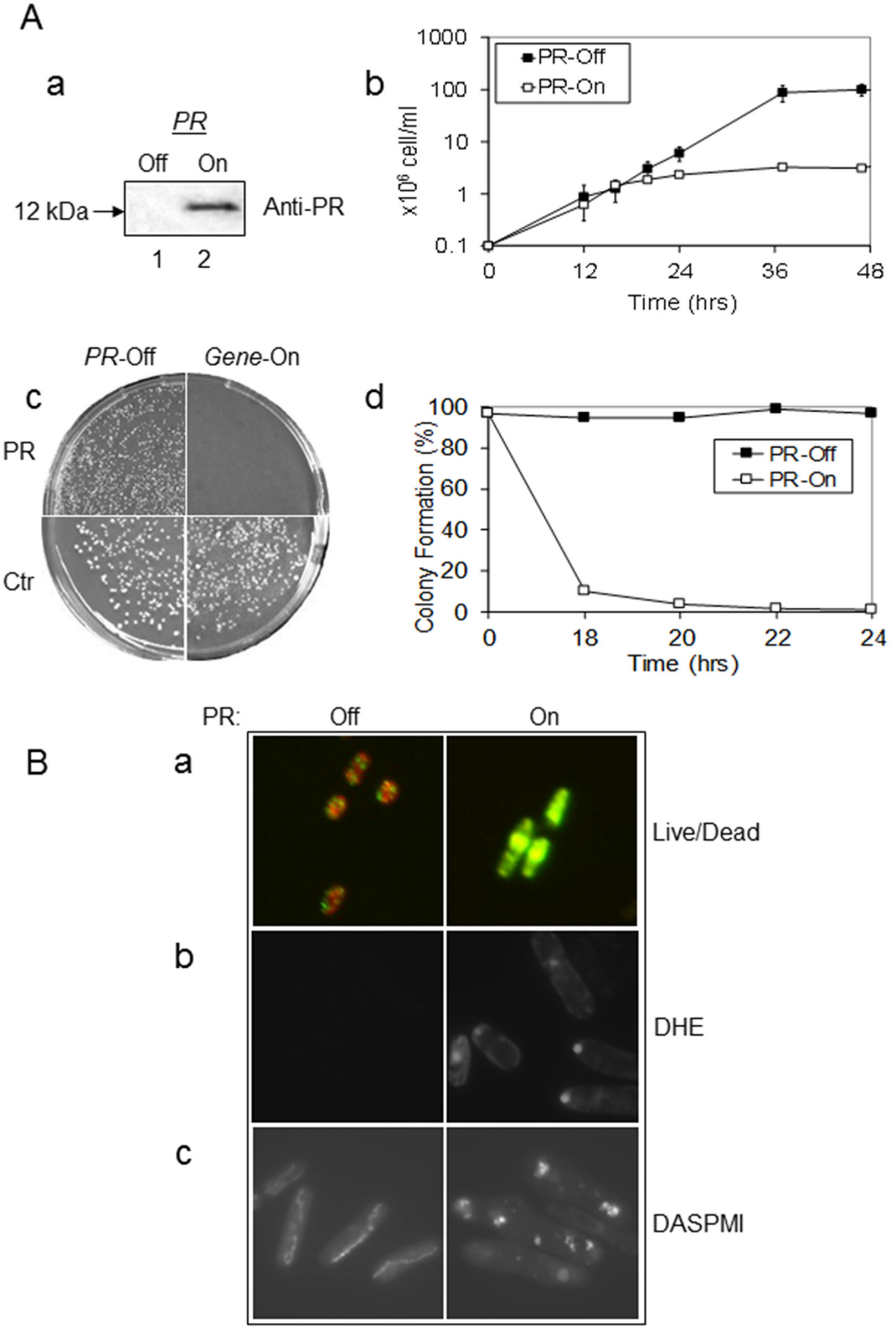
Expression of the HIV-1 *PR* gene prevents cellular growth and causes oxidative stress, changes of mitochondrial morphology, and cell death in fission yeast. (**A**) Inducible expression of the HIV-1 *PR* gene in RE294 was detected 24 hrs after gene induction by western blot analysis (**a**). Lane 1, *PR*-repressing cells; lane 2, *PR*-inducing cells. Blot shows a 12-kDa protein band that specifically reacted to an antiserum against HIV-1 PR. Expression of the *PR* gene induced cellular growth arrest in liquid medium (**b**) and prevented yeast colony formation on agar plates (**c**) and in liquid medium (**d**). *PR*-off, *i*.*e*., no PR protein production. *PR*-on, *i*.*e*., PR protein production. All cells were grown at 30°C, and the cell growth was measured at OD_650_ in the time period shown using a spectrophotometer. Pictures of agar plates were taken 6 days after incubation at 30°C under the indicated conditions. Error bars shown in (**b**) of the growth assay represent at least three independent experiments. (**B**) HIV-1 *PR* expression induced cell death (**a**), oxidative stress (**b**), and mitochondrial morphological changes (**c**) in fission yeast. Twenty-four hours after inducible *PR* expression, cell viability was measured by the yeast live/dead assay [30, 33] (**a**). In this assay, viable cells are typically shown in orange-red color (left, *PR*-off); metabolically deceased cells are shown in green-yellow color (right, *PR*-on). The production of reactive oxygen species (ROS) was measured by an ROS indicator dye DHE (**b**). Mitochondrial morphologies (**c**) were visualized by staining fission yeast cells with a mitochondria-specific fluorescent probe, DASPMI, as previously described [22, 36]. Note that normal mitochondria appear like a thread or necklace of multiple small dots concentrated around the edge a cell, at the growing ends of the cell, or as a tubular network extended along the periphery of the cell (Fig 1B-b, left). In contrast, different sizes of mitochondrial aggregates that are situated almost randomly throughout the *PR*-expressing cells are shown here, indicating changes in mitochondrial morphology (Fig 1B-b, right).

In one of our early genome-wide characterization studies of HIV-1 in fission yeast [31], HIV-1 *PR* appeared to affect cellular growth, suggesting that PR might be functional in fission yeast. Thus, the objective of this experiment was to verify this possibility. To carry out this experiment in a stable and physiologically relevant environment, a fission yeast strain RE294 was created that carried a single integrated copy of the HIV-1 *PR* gene in its chromosome. Expression of this gene was under the control of an inducible *n**o* *m**essage in* *t**hiamine 1* (*nmt1*) promoter. (For details of RE294 construction, see Supplementary Material S1 Fig). Specifically, in the presence of thiamine, the *nmt1* promoter is repressed (*gene*-off), and when thiamine is removed from the growth medium, the promoter is fully activated (*gene*-on) in about 14 to 16 hr [29, 32]. Indeed, as shown by western blot analysis in Fig 1A-a, a 12 kDa protein band, which reacted specifically to an antiserum against HIV-1 PR, was clearly shown 24 hrs after inducible expression of the HIV-1 *PR* gene (Lane 2), whereas no HIV-1 PR was seen in the *PR*-repressing cells (Lane 1). Consistent with the timeframe of inducible HIV-1 *PR* gene expression, cells initially showed non-distinguishable growth kinetics between *PR*-off and *PR*-on cell cultures; at 16 hrs after *PR* gene induction, however, the *PR*-off cells continued to grow exponentially, while the PR-producing cells stopped (Fig 1A-b).

In our earlier report [31], we observed that overexpression of HIV-1 *PR* prevented yeast colony formation. Here, we tested whether a single copy and stable expression of HIV-1 *PR* in the yeast chromosome could prevent yeast colony formation on an agar plate. As shown in Fig 1A-c, in the *PR*-suppressive medium, normal size colonies were seen (left), but when the same amount of RE294 cells were plated on the *PR*-inducing medium, few or no colonies were observed. In contrast, yeast colonies were formed under both gene-off and gene-on conditions when an empty pYZ1N vector was expressed, suggesting the removal of thiamine has no effect on colony formation. To further quantify the PR effect on colony formation, the same experiment was repeated over time in liquid medium (Fig 1A-d). Briefly, RE294 cells were grown in minimal medium with (*PR*-off) or without (*PR*-on) thiamine. An aliquot of the culture was collected at the indicated time and plated onto the thiamine-containing agar plates. The percentage of cells that formed colonies was calculated from the number of colonies that grew from the *PR*-on culture over the number of cells originally plated. Results showed that nearly 100% of the *PR*-off cells formed colonies, whereas less than 20% of the *PR*-on cells formed colonies 18 hrs after gene induction, suggesting a strong correlation between the timing of *PR* gene expression and the inhibition of yeast colony formation.

### Expression of HIV-1 *PR* induces cellular oxidative stress, changes in mitochondrial morphology, and cell death in fission yeast

We next tested whether prolonged expression of HIV-1 *PR* affected fission yeast cell viability. A commercial yeast live/dead viability assay was used to determine the status of intracellular metabolism [30, 33]. As shown in Fig 1B-a, when no PR was produced in fission yeast, cells showed an orange-red color suggesting viable and actively respiring cells (Fig 1B-a, left). However, 24 hrs after the HIV-1 *PR* gene was induced, fission yeast cells turned a greenish-yellow (Fig 1B-a, right), indicating cells were metabolically inert. These observations suggest that prolonged expression of HIV-1 *PR* induces cell death in fission yeast.

To explore the molecular mechanism underlying HIV-1 PR-induced cell death, possible intracellular stress induced by HIV-1 PR was determined by the production of oxidative stress species (ROS) [22]. A ROS-specific dye, dihydroethidium (DHE), which produces red fluorescence in the presence of ROS, was used to measure cellular oxidative stress in *PR*-expressing cells. As shown in Fig 1B-b (right), 24 hrs after *PR* gene induction, strong red fluorescence was detected in the *PR*-expressing cells, whereas little or no red fluorescence was observed in the *PR*-repressing cells (Fig 1B-b, left), suggesting HIV-1 PR indeed induced ROS in fission yeast.

Since HIV-1 PR induces apoptosis by interrupting mitochondrial functions in mammalian cells [14, 34] and in fission yeast, and because changes in mitochondrial morphology have also been correlated with an apoptosis-like process [22], we were interested to see whether PR has any effect on the mitochondrial morphology of fission yeast cells. Morphology of fission yeast mitochondria can be visualized by staining with a mitochondria-specific dye, 2-(4-dimethylaminostyryl)-1-methylpyridinium iodide (DASPMI) [22, 35]. Consistent with previous descriptions of normal fission yeast mitochondrial morphologies [22, 36], mitochondria in the *PR*-repressing cells (Fig 1B-c, left) appeared as tubular networks extending along the periphery of the cell. In contrast, mitochondria aggregated almost randomly in the *PR*-expressing cells (Fig 1B-c, right), indicating abnormal mitochondrial morphologies. Therefore, PR indeed alters mitochondrial morphology in *S*. *pombe* cells in ways that may correlate with PR-induced cell death and apoptosis.

### The protease inhibitor Indinavir suppresses HIV-1 PR-induced growth inhibition and cell death in fission yeast

**Fig 2:**
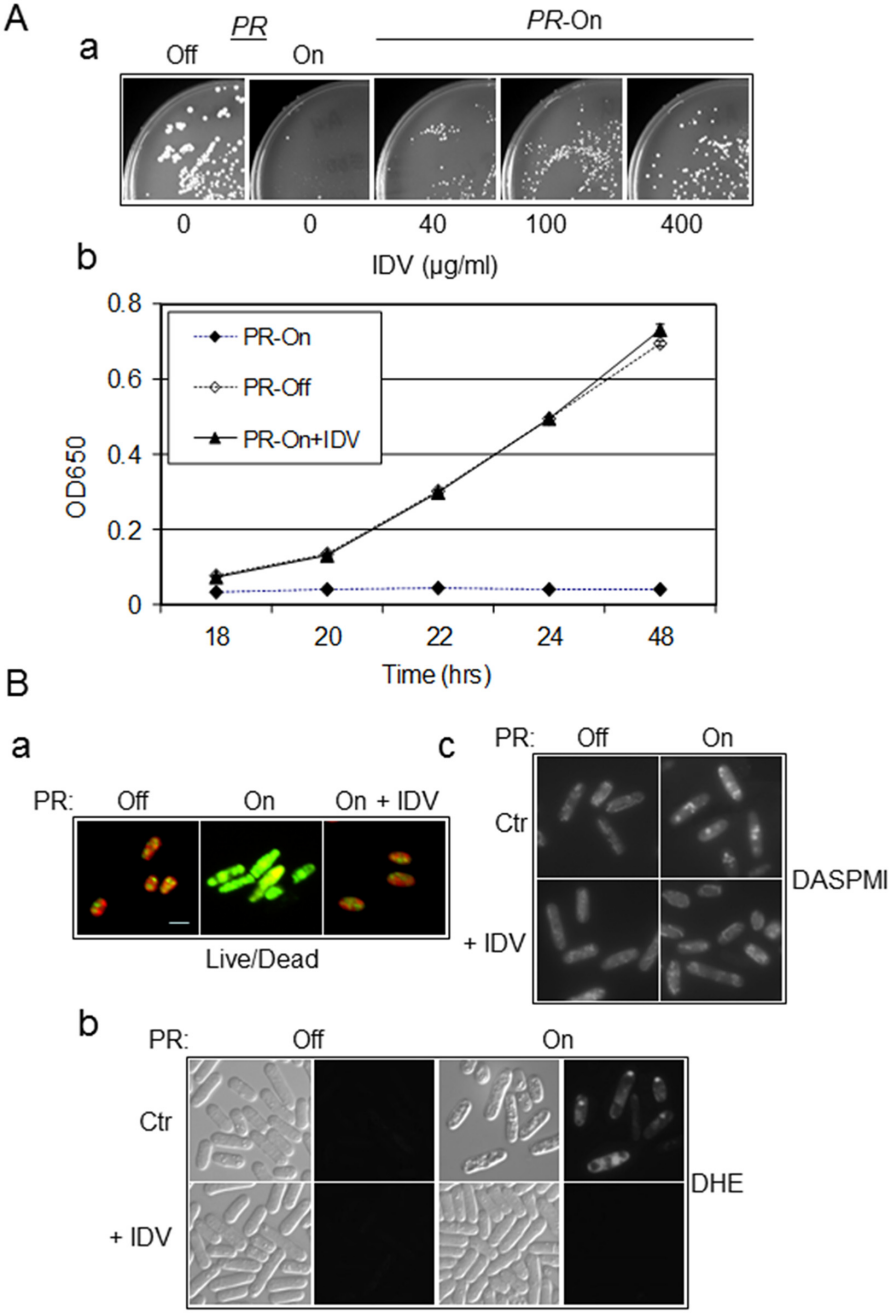
Growth inhibition and cell death induced by HIV-1 PR can be circumvented by IDV in fission yeast. (**A**) Treatment of HIV-1 *PR*-expressing fission yeast cells with protease inhibitor IDV restored colony formation in a dose-dependent manner (**a**) and cellular growth over time (**b**). The concentration of IDV that was added in (**a**) is shown, and 100 μg/mL of IDV was added to the *PR*-expressing cells in (**b**). All cells were grown at 30°C and cell growth was measured by OD_650_ at the time point indicating using a spectrophotometer. Error bars shown in (**b**) of the growth assay represent at least three independent experiments. **(B**) IDV prevents HIV-1 PR-induced oxidative stress, changes in mitochondrial morphology, and cell death. (**a**) Fission yeast cell death induced by HIV-1 PR, as shown by the yeast live/dead assay [30, 33], was prevented by adding 100 μg/mL of IDV before gene induction. Pictures were taken at 24 hrs after gene induction. ROS production (**b**) was detected and mitochondrial morphology (**c**) was observed using DHE and DASPMI as described in Fig 1. Ctr, control, *i*.*e*., no IDV added; + IDV, 100 μg/mL of IDV was added prior to HIV-1 *PR* gene induction.

To test whether the observed inhibitory effect of HIV-1 PR on cellular growth and colony formation was due to HIV-1 PR enzymatic activity, an FDA-approved HIV-1 PR inhibitor drug, IDV (or Crixivan^™^), which specifically inhibits enzymatic activity of PR, was used to test whether it could reverse the growth inhibition observed in fission yeast expressing HIV-1 PR. As shown in Fig 2A-a, when IDV was added to the *PR*-on agar plates at increasing concentrations between 40 and 400 μg/mL, colony formation was indeed restored, with the colony sizes approaching the normal size. Consistently, normal cellular growth was also completely restored when 100 μg/mL of IDV was added to the liquid PR-producing media (Fig 2A-b).

To further ascertain whether treatment of *PR*-expressing cells with IDV could prevent HIV-1 PR induced oxidative stress, changes in mitochondrial morphology, and cell death, the same experiments as described in Fig 1B were conducted. The same concentration of IDV (100 μg/ml) as described above was added to the HIV-1 *PR*-expressing cells before gene induction. Twenty-four hours after gene induction, IDV had completely reversed the effects of HIV-1 PR on ROS production, mitochondrial morphology, and cell viability (Fig 2B), suggesting that the observed inhibitory and cytotoxic effects of HIV-1 PR are most likely due to its proteolytic activities in fission yeast.

### Target-specific enzymatic protein cleavage of indigenous viral proteins by HIV-1 PR in fission yeast

**Fig 3:**
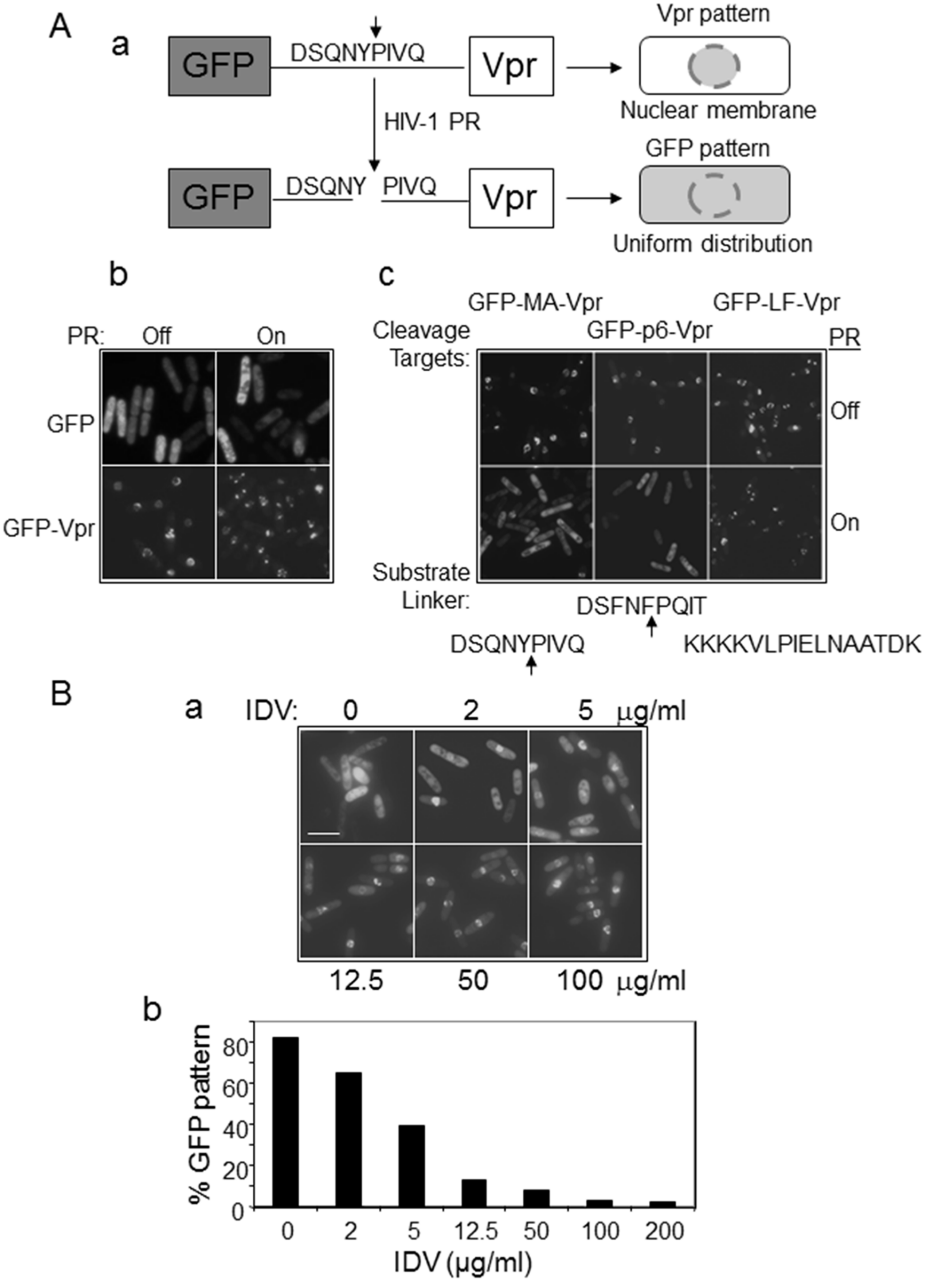
Target-specific enzymatic protein cleavage of indigenous viral proteins by HIV-1 PR and inhibition by IDV in fission yeast. **(A**) HIV-1 PR specifically cleaves GFP-p6/MA-Vpr fusion protein constructs that contain indigenous cleavage sites of HIV-1 p6 or MA. (**a**) Schematic drawing to show the rationale for tests of the proteolytic substrate specificity of HIV-1 PR in fission yeast. Green fluorescent protein (GFP) was distributed uniformly throughout fission yeast cells, i.*e*., the “GFP pattern” [39, 65]. HIV-1 viral protein R (Vpr) localizes predominantly to the nuclear membrane and appears as a “ring-like” structure, *i*.*e*., the “Vpr pattern” [39, 65]. (**b**) Expression of HIV-1 *PR* in fission yeast did not affect subcellular locations of GFP or HIV-1 Vpr-attached GFP. The top panel shows expression of GFP only; the bottom panel shows Vpr-attached GFP. (**c**) HIV-1 PR cleaves substrate linkers of HIV-1 MA (left) and p6 (middle), but not of LF (right). Cells were examined 20 hrs after *PR* gene induction. **(B**) IDV blocked the formation of the “GFP pattern” in a dose-dependent manner in fission yeast. The GFP-p6-Vpr fusion product was used here. The IDV effect was measured 20 hrs after gene induction at increasing concentrations as shown in (**a**). The percentage of cells exhibiting the GFP pattern, *i*.*e*., the putative protein cleavage of GFP-p6-Vpr fusion construct by HIV-1 PR, was quantified and is shown in (**b**). An average of 100–200 cells was counted for each concentration. Scale bar: 10 μm.

In order to verify whether PR indeed functions as a protease and exhibits the same enzymatic activities in fission yeast as it does in HIV-1 infection of mammalian cells, we developed a “green fluorescent protein (GFP) re-localization assay” that allowed us to specifically measure proteolytic activities of HIV-1 PR in fission yeast. In this assay, two GFP-p6/MA-Vpr gene fusion constructs were generated (Fig 3A-a), with each encoding, from the 5′ end, a *GFP* gene for fluorescent detection, a short p6 or MA polypeptide containing a known HIV-1 PR enzymatic cleavage site [37, 38], and an HIV-1 *vpr* gene encoding the Vpr protein that predominantly localizes to the nuclear membrane of fission yeast [39]. Two different HIV-1 PR cleavage sites in viral proteins MA and p6 were chosen for this study because the proteolytic target of HIV-1 PR is not entirely peptide-specific. Often, seemingly unrelated peptide sequences are cleaved and proteolyzed by HIV-1 PR, suggesting that recognition might be based on geometric specificity, determined by residues lying in a specific position to make the active target site accessible for cleavage [37]. Therefore, taking this target variability into consideration, an HIV-1 MA polypeptide linker (DSQNY↓PIVQ) that contained a natural PR cleavage site between Y and P was included in the GFP-MA-Vpr construct (Fig 3A-c, left) [37, 38]. Similarly, a different HIV-1 p6 polypeptide linker (DSFNF↓PQIT) was included in the GFP-p6-Vpr construct (Fig 3A-c, middle panels). As a control, a similar gene fusion construct (GFP-LF-Vpr) was created with a different protease cleavage substrate (KKKKVLPIELNAATDK) placed between GFP and Vpr that is specifically cleaved by an anthrax protease [40]. Therefore, by design, without PR, production of these fusion proteins in fission yeast should produce a “ring-like” structure on the nuclear membrane because of Vpr, *i*.*e*., the “Vpr pattern” (Fig 3A). Conversely, separation of GFP from Vpr as the result of PR cleavage at the substrate polylinker site will lead to the “GFP pattern,” with uniform distribution throughout the cell [39]. To make sure that expression of HIV-1 *PR* would not interfere with the subcellular distributions of GFP or Vpr, we examined the subcellular localizations of these two proteins with or without HIV-1 PR. As seen in Fig 3A-b, no significant changes were observed between the GFP and GFP-attached Vpr distributions in the presence or absence of PR, *i*.*e*., GFP was still dispersed throughout the cells and GFP-attached Vpr was primarily seen on the nuclear membrane under both conditions. Thus, we concluded that HIV-1 PR by itself does not affect the intracellular localization of GFP or Vpr in fission yeast. Therefore, we were able to measure the specific enzymatic activities of HIV-1 PR and substrate cleavages as designed.

Indeed, as expected, all three fusion proteins showed the “Vpr pattern” when no HIV-1 PR was present (Fig 3A-c, upper rows). However, when the HIV-1 *PR* gene was expressed 20 hours after gene induction, PR clearly cleaved the HIV-1 MA polypeptide linker (DSQNY↓PIVQ) in the GFP-MA-Vpr construct. The results showed that separation of Vpr from GFP led to re-localization of GFP from the “Vpr pattern” to the “GFP pattern” (Fig 3A-c, bottom left). A similar transition from the “Vpr pattern” to the “GFP pattern” was also seen in the GFP-p6-Vpr fusion protein (Fig 3A-c, bottom middle). In contrast, the distribution of GFP remained in the “Vpr pattern” with or without *PR* gene expression in the GFP-LF-Vpr construct, suggesting that PR did not cleave the LF site (Fig 3A-c, right row).

To further confirm that the observed HIV-1 PR enzymatic activity was the same as that during HIV-1 infection, the HIV-1 PR-specific enzymatic inhibitor IDV was used to block the proteolytic HIV-1 PR activities in the GFP-p6-Vpr construct (Fig 3B). As shown in Fig 3A, a clear “GFP pattern” was seen in the *PR*-expressing RE294 cells when no IDV was added (Fig 3B-a, top left), indicating that PR-mediated cleavage of GFP-p6-Vpr led to separation of GFP from Vpr. However, the green fluorescence became more and more concentrated on the nuclear membrane with increasing concentrations of IDV, from 2 μg/mL, 5 μg/mL, 12.5 μg/mL, and 50 μg/mL to 100 μg/mL (Fig 3B-a). When the percentage of the “GFP pattern” was determined and compared with the different doses of IDV, a significant and gradual decrease in the “GFP pattern,” an indication of reducing PR enzymatic activity, correlated with the increase in IDV dosage, suggesting dose-dependent inhibition of HIV-1 PR enzymatic activities by IDV (Fig 3B-b). Together, these data suggested that HIV-1 PR most likely displays the same enzymatic activities in fission yeast as it does in mammalian cells.

### The Hhp2 kinase is a novel PR suppressor that suppresses HIV-1 *PR*-induced protease cleavage and cell death in fission yeast

**Fig 4:**
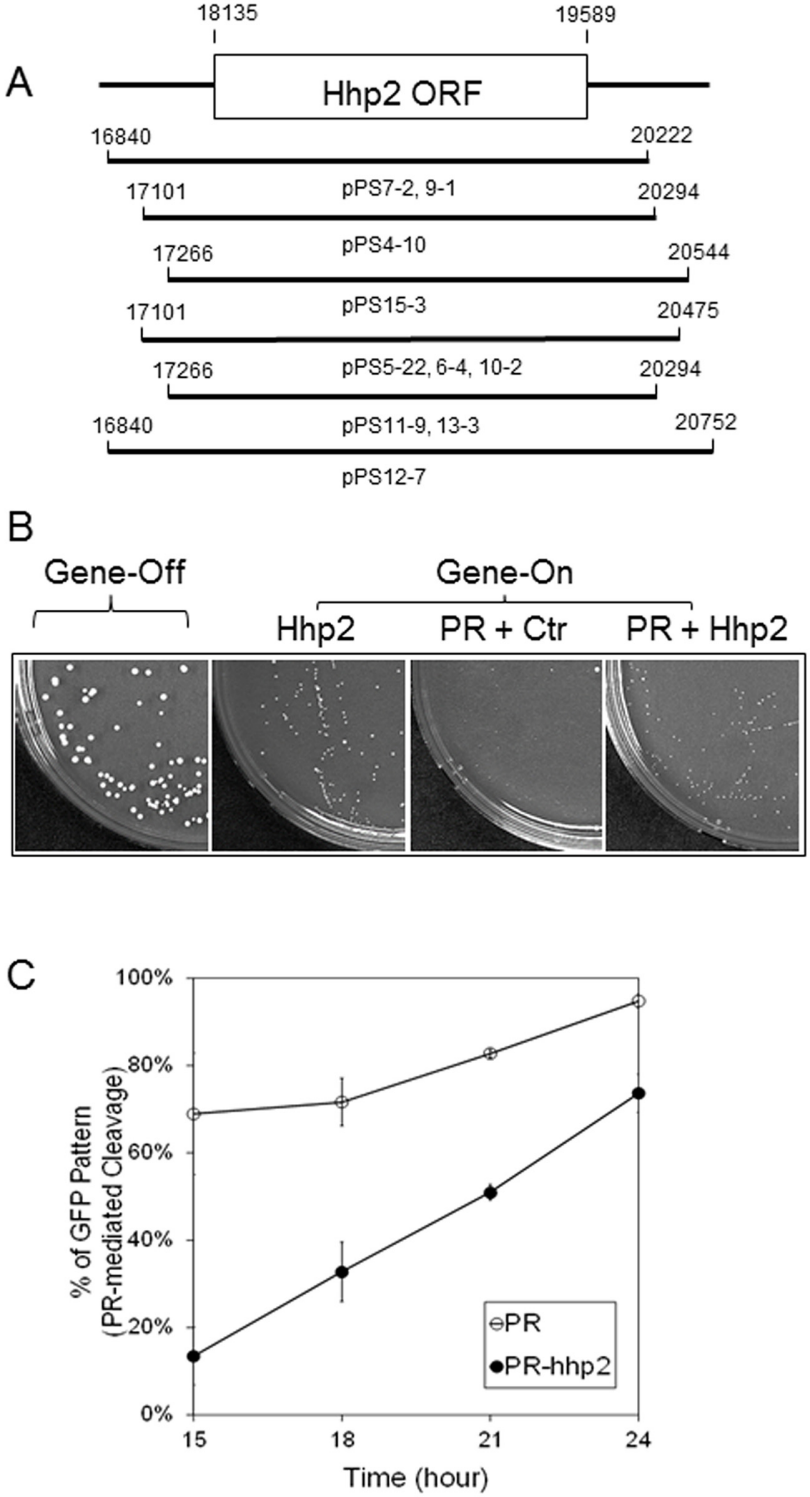
A fission yeast protein kinase Hhp2 suppresses PR activities. (**A**) Fission yeast genome-wide searches for multicopy suppressors of PR-induced cell death of fission yeast cells revealed six unique cDNA clones out of ten isolates with overlapping open reading frame (ORF) of the *hhp2* gene. The numbering and relative positions of each nucleotide were derived from the SPAC23C4 cosmid of fission yeast. (**B**) Overexpression of *hhp2* restored colony formation in *PR*-expressing fission yeast cells. Expression of the *hhp2* gene was induced at lower levels with 1 nM thiamine added to the media. Under this condition, HIV-1 PR still prevented colony formation as shown. Ctr, an empty pYZ1N plasmid control. *Gene*-off, no HIV-1 PR or Hhp2 protein production; *Gene*-on, *i*.*e*., PR protein production in the presence of Hhp2 or an empty plasmid vector. Agar plates were incubated at 30°C under the indicated conditions for 6 days before pictures were taken. (**C**) Overexpression of *hhp2* reduced the percentage of cells showing the GFP pattern over time. The GFP-p6-Vpr fusion product was used here. The Hhp2 effect was measured over time after gene induction as indicated. The percentage of GFP pattern, *i*.*e*., the putative protein cleavage of GFP-p6-Vpr fusion construct by HIV-1 PR was quantified and is shown in (**b**). Error bars shown in (**C**) represent results of at least three different experiments with an average of 100–200 cells counted at each time point.

In order to explore the molecular interactions of HIV-1 PR with cellular proteins, we searched for possible fission yeast PR-suppressing proteins (PSPs) by overexpressing fission yeast genes in the *PR*-carrying RE294 strain. Specifically, a genome-wide screen was launched using a fission yeast genomic cDNA library in RE294 cells expressing the *PR* gene. In principle, a PSP could be identified by its suppressive activity against HIV-1 PR, leading to formation of yeast colonies on a *PR*-inducing agar plate. At least 40,000 transformants, which statistically covers the entire fission yeast genome, were screened. Eleven possible suppressor candidates were identified. Their abilities to suppress HIV-1 PR were confirmed by reintroducing the corresponding plasmids back into the parental RE294 fission yeast strain. Ten of the eleven plasmids could suppress the PR effect, with one being a false positive. When those ten plasmids were sequenced, all of them carried the *hhp2* gene, with 6 unique cDNA fragments containing different lengths of gene inserts (Fig 4A). The *hhp2* gene encodes a protein that is made of 400 amino acids with a molecular weight of approximately 45.83 kD. It belongs to the casein kinase 1 (CK1) class of serine/threonine kinases [41].

The original suppressing strength of Hhp2 against HIV-1 PR was noted to be quite weak, because relatively small colonies were seen on agar plates with co-production of HIV-1 PR and Hhp2. To test whether higher levels of *hhp2* gene expression could enhance the suppressive effect, the *hhp2* gene was cloned into plasmid pYZ1N and overexpressed in RE294. However, full expression of *hhp2* under the *nmt1* promoter was toxic to cells (data not shown). Thus, we had to reduce expression of *hhp2* in subsequent suppression studies by adding 1 μM thiamine to the growth media. Under this condition, as shown in Fig 4B, the Hhp2-producing cells formed smaller colonies than the normal (gene-off) cells. Importantly, HIV-1 PR was still able to block colony formation (Fig 4B, the second plate on the right), thus allowing us to test the suppressive effect of Hhp2 on PR. Consistent with what we observed during the initial genome-wide screenings, when PR and *hhp2* were co-expressed, instead of seeing no colonies on the PR-producing agar plates, small yeast colonies were seen (Fig 4B, right plate), suggesting that Hhp2 restored colony formation by PR-producing yeast cells at least in part.

Under the same *hhp2* expressing condition, we tested whether Hhp2 could alleviate the proteolytic activity of HIV-1 PR. The same GFP-p6-Vpr fusion construct as shown in Fig 3B was used to measure the percentage of cells exhibiting the “the GFP pattern”, an indication of PR-mediated cleavage, over time in the presence of Hhp2. As shown in Fig 4C, without Hhp2, PR cleavage increased from about 70% to nearly 100% by 24 hrs after *PR* gene induction. Significantly, about a 20–50% reduction in PR cleavage was observed during the same time period when the *hhp2* gene was co-expressed. Together, the data presented here showed that we identified a fission yeast Hhp2 kinase that is a novel PR suppressor and that specifically suppresses HIV-1 PR-induced protease cleavage and cell death in fission yeast.

### Hhp2 kinase suppresses HIV-1 *PR*-induced cell death and apoptosis in mammalian cells

**Fig 5:**
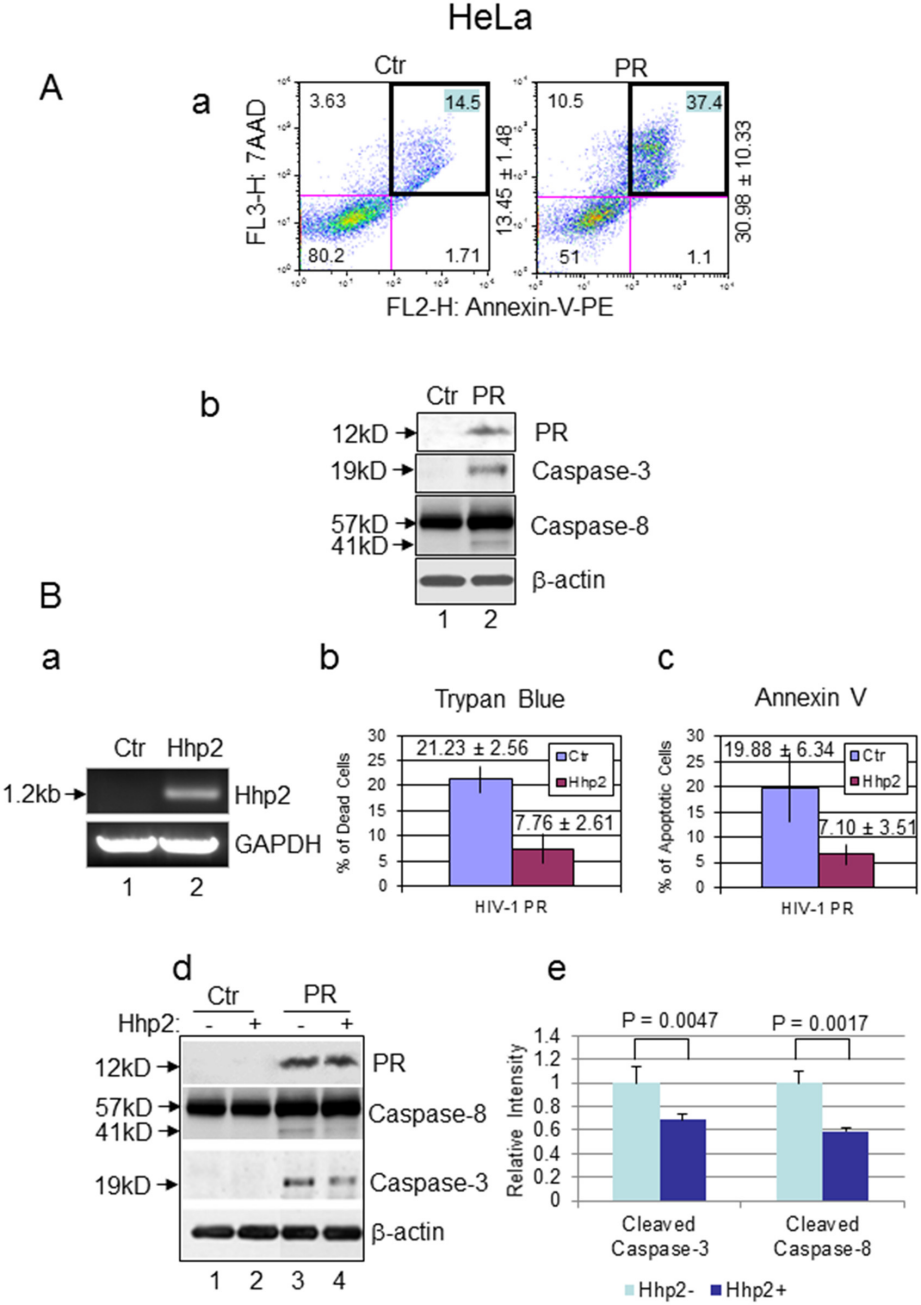
Hhp2 suppresses *PR*-induced cell death and apoptosis in HeLa cells. **(A)** Expression of HIV-1 *PR* induces apoptosis in HeLa cells. Apoptosis were detected by staining with Annexin V and FACScan analysis as previously described [64]. The percentage of apoptotic cells was measured in HeLa cells 24 hrs after transfection of a control (Ctr) pIRES2-EGFP plasmid (left) or a *PR*-containing pIRES2-EGFP-*PR* plasmid (right). The average number ± SD of apoptotic cells are shown in the top right quadrant (**a**), using data from at least three independent measurements. (**b**) Expression of HIV-1 PR induced caspase-3 and caspase-8 cleavage in HeLa cells as shown by western blot analyses performed as previously described [25]. Lane 1, HeLa cells transfected with a control plasmid; lane 2, *PR*-expressing transfected HeLa cells. The analysis was carried out 48 hrs after plasmid transfection. (**B**) Hhp2 suppresses *PR*-induced cell death and apoptosis in HeLa cells. Since no specific antibody against Hhp2 was available, to confirm *hhp2* gene expression in HeLa cells, *hhp2* gene transcription was measured by RT-PCR and detected on agarose gel as shown in (**a**). Lane 1, HeLa cells transfected with a control plasmid; lane 2, *hhp2*-expressing HeLa cells. The gel shows the 1.2-kb *hhp2* cDNA that was amplified. Glyceraldehyde 3-phosphate dehydrogenase (GAPDH) was used as an internal control. The suppressive effect of Hhp2 on HIV-1 PR-induced cell death and apoptosis was also quantified 24 hrs after gene expression using a Trypan blue assay (**b**) and Annexin V staining (**c**), respectively. The average numbers ± SD of dead cells are shown, the error bars represent results of at least three independent measurements. The effect of Hhp2 on HIV-1 PR-mediated caspase-3 and caspase-8 cleavage was detected by western blot analysis as shown in (**d**) and quantified in (**e**). A statistical t-test was used to determine whether there was a significant difference in the cleavage of caspase-3 and caspase-8 taking place in the control HeLa cells and HIV-1 *PR*-expressing cells, with or without Hhp2. p-values less than 0.01 were considered statistically significant.

Previous studies have showed that HIV-1 PR induces apoptosis in CD4+ mammalian T-cells [42, 43]. One of the unique features of PR-induced apoptosis is that it cleaves caspase-8 and generates a distinctive fragment of casp8p41, which in turn leads to stimulation of an apoptotic signaling cascade that involves loss of mitochondrial transmembrane potential and activation of caspase-9 and caspase-3 [42, 43]. Thus casp8p41 is specific to HIV-1 *PR*-induced cell death of infected CD4^+^ T cells. To test whether HIV-1 PR induces apoptosis in HeLa cells and shows the same unique features as in CD4+ T cells, we tested the effect of HIV-1 PR by expressing *PR via* a CMV promoter carried on the pIRES2-EGFP plasmid. Production of the PR protein in HeLa cells was first confirmed by western blot analysis. PR-induced apoptosis was then examined by Annexin V staining and detected by flow cytometry and the caspase cleavages were confirmed by western blot analysis. As shown in Fig 5A-a, 24 hrs after *PR* gene expression, the percentage of apoptotic cells (top right quadrant) increased significantly from 13.45 ± 1.48% to 30.98 ± 10.33% in comparison with that of the vector control, suggesting that HIV-1 PR indeed causes apoptosis in HeLa cells.

Since casp8p41 is specific to HIV-1 *PR*-induced cell death of infected CD4^+^ T cells [43], we next tested whether HIV-1 PR could also induce a similar casp8p41 protein product in HeLa cells. Possible cleavages of caspase-8 and caspase-3 were detected by western blot analysis. As shown in Fig 5A-b, following *PR* gene expression, a relatively weak protein band right underneath capase-8 with a molecular weight of approximately 41 kD appeared in the PR-producing cells (Lane 2), whereas no cleaved protein band was seen underneath capase-8 of the control cells (Lane 1). Note that even though the 41-kD protein band was relatively weak, suggesting low cleavage efficiency of its precursor, production of this protein band was reproducible. Consistently, a cleaved product of caspase-3 was also seen in the PR-producing cells, whereas no capase-3 cleaved product was observed in the control HeLa cells. Together, these data suggest that HIV-1 PR also induces cell death and apoptosis in HeLa cells with the same characteristic generation of casp8p41.

Because fission yeast Hhp2 suppresses *PR*-induced cell death in fission yeast, we were interested in testing whether Hhp2 could also suppress *PR*-induced cell death and apoptosis in HeLa cells. The *hhp2* gene was cloned into the mammalian expression vector pcDNA3.1 and expressed in HeLa cells with selection by hygromycin B. Because there are no specific antibodies against yeast Hhp2, expression of *hhp2* gene in HeLa cells was first confirmed by measuring *hhp2* mRNA transcripts in an RT-PCR analysis (Fig 5B-a). HeLa cells were then transfected with the *PR*-expressing vector pIRES2-EGFP-PR. As a control, an equal amount of HeLa cells were transfected with an empty pIRES2-EGFP plasmid. The effect of *hhp2* on *PR*-induced cell death was evaluated by staining cells with Trypan blue (Sigma-Aldrich). Trypan blue-negative cells are viable cells, whereas Trypan blue-positive cells are dead cells [44]. To measure the Hhp2 effect on cell death, the percentage of dead cells were calculated by counting the numbers of blue cells over the total cells. As shown in Fig 5B-b, 24 hrs after gene expression, HIV-1 PR killed about 21.23 ± 2.56% of the HeLa cells. In contrast, 7.76 ± 2.61% dead cells were detected in the presence of Hhp2. A very similar suppressive effect of Hhp2 on PR-induced apoptosis (19.88 ± 6.34% *vs*. 7.10 ± 3.51%) was also observed when those cells were stained with Annexin V (Fig 5B-c). Consistent with the suppressive effect of Hhp2 on HIV-1 PR-induced cell death and apoptosis, the western blot analyses further showed that expression of *hhp2* in the PR-producing HeLa cells also reduced the cleavage levels of caspase-3 and generation of casp8p41 (Fig 5B-d, lane 4 *vs*. lane 3). A quantitative comparison showed that there was a statistically significant reduction in the cleaved caspase-3 and casp8p41 products of the HIV-1 *PR*-expressing cells with and without Hhp2, compared to the control (Fig 5B-e). Therefore, the combined data suggest that fission yeast Hhp2 kinase indeed suppresses, at least in part, HIV-1 PR-induced cell death and apoptosis in HeLa cells.

## Discussion

In this study, we demonstrated for the first time that HIV-1 protease is functional in fission yeast and behaves in a similar manner as it does in mammalian cells. Specifically, HIV-1 PR proteolyzed two HIV-1 viral proteins (p6 and MA) at the same substrate cleavage sites as it does in HIV-1 infection of mammalian cells (Fig 3A) [38], whereas it did not proteolyze the LF protease substrate of *Bacillus anthracis* [40]. The fact that HIV-1 PR specifically proteolyzed two different types of indigenous HIV-1 viral substrates in fission yeast suggests that the observed cleavage of GFP-p6/MA-Vpr were indeed due to the enzymatic activities of HIV-1 PR. This notion was certainly supported by the fact that both of these PR-mediated protein cleavages were prevented by a PR-specific enzymatic inhibitor, IDV (Fig 3B).

Similar to the PR-induced cell death and apoptosis in mammalian cells, production of PR in fission yeast prevented cell proliferation and caused cell death in a manner that was reminiscent of apoptosis (Fig 1) [22, 27]. This showed that HIV-1 PR not only triggered ROS production, an indication of oxidative stress, but that it also caused changes in mitochondrial morphology that are linked to apoptosis [22, 36]. Indeed, we further showed that, when the HIV-1 *PR* gene was expressed in HeLa cells, PR also induced cell death and apoptosis as previously reported [43, 45–47]. Consistent with what was shown in infected CD4+ T cells, HIV-1 PR also cleaved capase-8 and produced the characteristic casp8p41 fragment in HeLa cells (Fig 5A-b and 5B-d) [13, 43]. Interestingly, a recent report showed that HIV-1 PR localizes in mitochondria, where it interacts with a cellular protein, breast carcinoma-associated protein 3 (BCA3), and triggers apoptosis by proteolytic cleavages of the mitochondrial proteins Tom22, VDAC, and ANT, leading to the release of apoptosis-inducing factor and a decrease in the mitochondrial membrane potential [14]. At present, the molecular mechanism underlying HIV-1 PR-induced cell death and apoptosis is still elusive. However, these cell killing activities are clearly due to interactions between HIV-1 PR and host cellular proteins, presumably because its proteolytic activities. This notion was clearly supported by our study showing that both PR-mediated protein cleavage and PR-induced cell death can be circumvented by the PR-specific enzymatic inhibitor IDV, suggesting that HIV-1 PR’s effects were probably mediated through its enzymatic activities.

The activities of HIV-1 PR have previously been described in budding yeast (*Saccharomyces cerevisiae*) [45]. In that study, the expression of HIV-1 *PR* arrested yeast growth that was followed by cell lysis. The lytic phenotype included loss of plasma membrane integrity and cell wall breakage, leading to the release of cell contents into the medium [45]. Interestingly, even though HIV-1 PR also prevented cell proliferation and induced cell death in fission yeast [31] (Fig 1), no clear cell lysis was observed. The differences between the two yeasts could potentially be explained, at least in part, by the relative thicker cell wall of fission yeast. Nevertheless, the observed cell death by cell lysis shown in budding yeast was probably due to the proteolytic activity of HIV-1 PR, because, in another study, a battery of HIV-1 protease inhibitors, including IDV, were used, showing that they indeed prevented cell lysis-induced cell death [48]. Different from the budding yeast study, however, we directly demonstrated that HIV-1 PR displays the same enzymatic activity as it does during HIV-1 infection of mammalian cells by cleavage its indigenous viral targets [37, 38] (Fig 3). Furthermore, we showed that HIV-1 PR-induced cell death in fission yeast resembles apoptosis of mammalian cells [22, 36], and both PR-induced proteolytic cleavage of protein targets and PR-induced cell death can be prevented by the specific PR inhibitor IDV (Figs 1B and 2B), Together, our data suggested that HIV-1 PR functions in a very similar fashion in fission yeast as it does in mammalian cells.

Through a protein-protein interaction analysis, HIV-1 PR has been shown to interact with many cellular proteins [9]. However, the functional relevance of those interactions is currently unknown. To explore possible functional interactions of HIV-1 PR with cellular proteins, we conducted a genome-wide screening for multicopy suppressors of *PR*-induced cell death and identified a protein kinase Hhp2 that, when it was expressed at the proper level, suppressed PR-induced cell death and protein cleavage in fission yeast (Fig 4). A similar suppressive effect of Hhp2 on PR-induced cell death and apoptosis was also observed in mammalian cells (Fig 5A). Hhp2 is a highly conserved serine/threonine kinase, which is one of the two isoforms of CK1 in fission yeast. It also shares significant sequence identities with budding yeast Hrr25p and human CK1α kinase [41, 49]. However, the molecular and suppressive action of Hhp2 toward HIV-1 PR is at present unknown. The fact that Hhp2 suppressed both PR-mediated protein cleavage and cell death/apoptosis suggests that Hhp2 might suppress HIV-1 PR directly. There are a number of possible molecular mechanisms that might underlie the suppressive effect of Hhp2 on HIV-1 PR. First, since Hhp2 is a kinase, Hhp2 could potentially inhibit the PR activities by phosphorylation. Although we have yet to find specific evidence of inhibition of HIV-1 PR by phosphorylation *via* Hhp2 or its mammalian homologs, another kinase, CK2, was shown to increase the PR phosphorylation *in vitro* [10]. Second, Hhp2 could inhibit HIV-1 PR by binding to PR directly. This is certainly possible, because HIV-1 PR binds to many cellular proteins, including BCA3 [9, 14]. The PR-BCA3 interaction elevates the level of pro-apoptotic Bax protein in cells, leading to HIV-1 PR-mediated apoptosis [14]. Finally, Hhp2 could be a direct cellular cleavage target of HIV-1 PR that induces cell death and/or apoptosis. Supporting evidence include reports showing that HIV-1 PR specifically cleaves other serine/threonine kinases, including the NDR1 and NDR1 kinases [11], receptor-interacting protein kinase 1 (RIPK1), and RIPK2 in CD4+ T cells during HIV-1 infection [12]. Most interestingly, interfering with the viral life cycle at different stages by the addition of specific inhibitors against PR completely prevented RIPK1 and RIPK2 cleavage. Since both of these two kinases contain conserved domains that are important for apoptosis, necrosis, and innate immunity, these findings indicate that RIPK1 and RIPK2 are targets for HIV-1 PR activity during HIV-1 infection and that their inactivation may contribute to cell death and modulation of host defenses by HIV-1 [12]. Conceivably, a similar interaction of HIV-1 PR with the mammalian homologs of Hhp2 could also be present in HIV-infected cells. Since we now know that Hhp2 is involved in PR-induced cell death and apoptosis, under this scenario, a mammalian homolog of Hhp2 could potentially be part of the cellular signaling pathway for PR-mediated induction of cell death and/or apoptosis in mammalian cells. Indeed, earlier reports have shown that targeted inhibition of CK1α, a mammalian homolog of Hhp2, by a CK1-specific inhibitor resulted in cell death [50, 51]. Thus, it would be interesting to test these possibilities in future studies.

Besides facilitating studies on the interactions of HIV-1 PR with cellular proteins as described in this study, the development of a fission yeast model system for HIV-1 PR could have additional advantages or applications. For example, fission yeast is a simple and unicellular organism with cellular functions that in many ways resemble those of mammalian cells [15, 16]. Thus, knowledge learned from this model organism about HIV-1 PR could allow new aspects of this viral protein to be uncovered that may not be readily revealed simply by studying mammalian cells. With this demonstration of the functional relevance of HIV-1 PR in fission yeast, high-throughput systems for future drug discovery against HIV-1 proteases, especially those that confer drug resistance to the current antiretroviral regimens, could also be developed. The advantages of using this type of cell-based high-throughput drug screening system include its cost-effectiveness, the ability to screen drugs in a non-infectious environment, and the ability to preclude those compounds that are toxic to cells.

## Materials and Methods

### Fission yeast strains and mammalian cells

A commonly used wild-type fission yeast (*S*. *pombe*) strain SP223 (*h-*, *ade6-216*, *leu1-32*, *ura4-294)* was used in this study [52]. RE294 is a derivative of SP223, which was generated during this study and contains a single integrated copy of the HIV-1 *PR* gene at the *nmt1* gene locus (See Supplementary Material S1 Fig). HIV-1 *PR* gene expression is under the control of an inducible no message in thiamine (*nmt1*) promoter and a *kan*^*r*^ gene adjacent to it confers resistance to the drug geneticin (G418). Specifically, gene expression can be fully induced or repressed in the absence or presence of 20 μM thiamine, respectively [32, 53]. Reduced gene expression can also be achieved by addition of thiamine at a less than 20 μM concentration (our unpublished data). Standard complete yeast extract with supplement (YES) medium, Edinburgh Minimal Medium (EMM), and Pombe Glutamate Medium (PMG) supplemented with adenine, uracil, leucine, thiamine (20 μM), or G418, when necessary, were used for yeast cell growth and plasmid selection. A protease inhibitor indinavir (IDV, Crixivan^™^), which is a FDA approved drug for antiretroviral therapy, was used at the concentrations indicated in this study to suppress HIV-1 PR enzymatic activities.

A human cervical epithetical HeLa cell line [21] was used to characterize HIV-1 PR activities in mammalian cells. HeLa cells were grown in Dulbecco’s modified Eagle’s medium (DMEM) supplemented with 10% fetal bovine serum (FBS).

### Plasmid vectors and molecular cloning

The fission yeast expression plasmid vectors pYZ1N or pYZ3N, which have been previously described [39, 54], were used in this study for gene expression studies. Both plasmids carry a thiamine repressible *nmt1* promoter, allowing the gene of interest to be expressed in an inducible manner. The plasmids pYZ1N and pYZ3N (for GFP-fusion) carry a *leu2* gene as a selection marker. A fission yeast genomic library (a gift from Dr. Anthony Carr), cloned into the plasmid pUR18 carrying an *ura4* selection marker [55], was used to screen for multi-copy suppressors of HIV-1 PR in fission yeast. The identified HIV-1 PR suppressor gene, *hhp2*, was subsequently re-cloned into the inducible gene expression vector pYZ1N. All yeast plasmid transformation was done by electroporation using the BTX Electro Cell Manipulator (ECM) 600 System protocol 0226 [56].

The mammalian gene expression plasmid pcDNA3.1 (Invitrogen), which carries a hygromycin B-resistant gene, was used to clone the fission yeast *hhp2* gene, and its expression in mammalian cells was selected under 200 μg/mL of hygromycin B. The HIV-1 *PR* gene, which was obtained from the HIV-1 carrying plasmid pNL4-3, was cloned into the mammalian expression vector pIRES2-EGFP (Clontech) that carries a neomycin resistance gene.

### Fission yeast assays

To measure cellular growth and gene induction in fission yeast cells, standard culture techniques were used [29, 57]. Briefly, all fission yeast cells were grown either in the minimal EMM or PMG media. Cells carrying plasmids with the *nmt1* promoter were maintained selectively in appropriately supplemented media with 20 μM thiamine to silence gene expression. For gene induction, cells were first grown to mid-log growth phase in the presence of 20 μM thiamine. Cells were then washed three times with distilled water and diluted to a final concentration of approximately 2 × 10^5^ cells/mL in 5 mL of appropriately supplemented EMM/PMG media with (gene-off) or without (gene-on) thiamine. All cells were routinely grown at 30°C with constant shaking of 250–300 rpm. Cell growth was measured at each time point either by manual counting of cell numbers or by automated measurement of the optical density (OD_650_) using a spectrophotometer.

A fission yeast colony-forming ability [58, 59] assay was used to investigate the effect of the *PR* gene expression on fission yeast cell proliferation and viability. Briefly, RE294 cells were prepared the same way as described above for yeast cell growth. An aliquot of *PR*-on or *PR*-off liquid culture was collected at the indicated time points after *PR* gene induction and was plated onto the thiamine-containing (*PR*-off) agar plates. The effect of PR on colony-forming ability was evaluated 6 days after plating by comparing the colony sizes between plates with or without PR production. An empty pYZ1N vector was also used as a control. The percentage colony formation at each time point was calculated from the number of colonies that grew from the *PR*-on culture as a percentage of the number of cells originally plated, which was further calibrated by the plating efficiency of the *PR*-off culture.

HIV-1 *PR*-induced cell death in RE294 was measured using a commercial live/dead yeast viability kit (Cat. No. L-7009; Invitrogen, Carlsbad, CA) [59–61]. Briefly, thiamine was removed from a logarithmic-phase cell culture as described above. The cells were then diluted to a concentration of 4 × 10^4^ cells/mL, and re-suspended in PMG minimal medium supplemented with or without thiamine to suppress or induce HIV-1 *PR* gene, respectively. The cell cultures were grown at 30°C with constant shaking at 300 rpm, collected at 24 hours, and resuspended in the GH solution (2% D-(+)-glucose +10 mM Na-HEPES, pH 7.2). A 50-μL aliquot of FUN-1 solution (80 μM) was added to an equal volume of cell suspension. The suspension was further incubated at 30°C for 45 minutes. About 3 μL of the suspension was applied onto a glass slide, covered with a coverslip, and sealed with wax. The cell viability status was examined using a Leica DM fluorescent microscope with a 11001v2 long path Chroma filter cube. Typically, actively respiring cells are marked clearly with orange-red fluorescent structures at a maximum wavelength of approximately 590 nm, whereas metabolically insert or dead cells exhibit bright, diffuse, green-yellow fluorescence at a maximum wavelength of approximately 540 nm [59, 60]. FUN1-stained cell images were collected at the excitation wavelength of 470 ± 20 nm with red, green, and blue filters set to generate color images by fluorescence merging.

Induction of cellular oxidative stress by HIV-1 PR was determined by the production of ROS, which was detected by an ROS-specific dye, dihydroethidium (DHE, Sigma) that produces red fluorescence in the presence of ROS as described previously [22, 31, 62]. Cells were grown as described above. Twenty-four hours after *PR* expression, DHE was added at a concentration of 5 μg/mL, and ROS were detected by fluorescence microscopy.

The mitochondrial morphology of fission yeast was visualized using a vital dye, 2-(4-dimethylaminostyryl)-1-methylpyridinium iodide (DASPMI, Sigma), as previously described [22, 36]. Cells and HIV-1 *PR* gene expression were induced for 24–36 hrs as described above. Prior to observation, DASPMI was added to the culture at a final concentration of 75 μg/mL. Cells were incubated at 36°C for 5 min, recovered by centrifugation in a microcentrifuge for 30 sec at 500 g, resuspended in 20 μL of YES, and then examined immediately with the fluorescence microscope (L5 filter) at an excitation wavelength of around 470 nm and emission wavelength of 560–570 nm.

### Fluorescence microscopy

A Leica fluorescence microscope DMR4500B equipped with a high performance CCD camera (Hamamatsu) and Open-Lab software (Improvision, Inc., Lexington, MA) was used for all imaging analyses. Fission yeast cells were collected onto a regular glass slide and covered with a cover slip. For the observation of green fluorescence, we used a Leica L5 filter with excitation of 480/40 and emission of 527/30. For red fluorescence, we used a Leica N2.1 filter with excitation of 537.5/22.5 and emission of LP590. To observe green-yellow fluorescence, we used a Leica YFP filter with excitation of 500/20 and emission of 535/30.

### Measurement of HIV-1 PR activity and substrate specificity in fission yeast

To test whether HIV-1 PR can recognize and cleave the same viral protein recognition sites in fission yeast as it does during HIV-1 infection of mammalian cells, we developed a GFP re-localization assay that allowed us to specifically measure proteolytic activities of HIV-1 PR. Briefly, two “GFP-p6/MA-Vpr” gene fusion constructs were generated in the fission yeast expression vector pYZ3N [39, 54], each encoding GFP for fluorescent detection, an HIV-1 PR enzymatic cleavage site that was derived from the HIV-1 MA-CA or the p6-PR cleavage sequence [38], and the HIV-1 Vpr protein that is predominantly localized to the nuclear membrane in fission yeast [39] (also see Fig 3A). Consequently, expression of the fusion protein without protease cleavage will appear predominantly as a “ring-like” structure on the nuclear membrane because of a property of Vpr, *i*.*e*., the “Vpr pattern” [39]. In contrast, separation of GFP from Vpr due to PR cleavage at the substrate site leads to the “GFP pattern,” with uniform distribution throughout the cell [39]. As a control, a similar fusion construct (GFP-LF-Vpr) was created with a polypeptide of anthrax lethal factor (LF) that contains a substrate of a *Bacillus anthracis* protease [40]. To examine PR enzymatic activity, the fission yeast cells were prepared as described above and collected 20 hrs after gene induction. The percentage of protein cleavage by HIV-1 PR (% of the GFP pattern) was calculated as the number of cells showing dispersed distribution of GFP throughout the cell (an indication of PR cleavage), *i*.*e*., the “GFP pattern” over the entire cell population.

### Genome-wide screening for multicopy suppressors of HIV-1 PR in fission yeast

A genome-wide search for multicopy suppressors of HIV PR was carried out by transforming a fission yeast expression cDNA library carried on the pUR18 plasmid [55] into the strain RE294. Fission yeast genes in the cDNA library were constitutively expressed from their own indigenous promoters, and modest overexpression was expected with the estimated plasmid copy number of 5–10 per cell. Expression of the *PR* gene in RE294 was under the control of an inducible *nmt1* promoter [32, 63]. To search for potential PR suppressors, at least 40,000 RE294 transformants containing plasmids that presumably cover all of the fission yeast cDNAs were spread on EMM agar plates deprived of thiamine (EMM-T). The criterion used to identify suppressors of HIV *PR*-induced cell death was the ability of a fission yeast transformant to form colonies on *PR*-inducing plate as previously described [21]. Therefore, colonies formed following gene expression were transferred to a YES agar plate supplemented with 200 mg/L of G418 to eliminate those cells that might have lost the *PR* gene (revertants). Cells that still maintained their colony forming ability were replicated on the EMM-T inducing media to retest their growth ability. Transformants that could still form colonies were identified as possibly having a plasmid that contained a PR suppressor in their cDNA insert. Lastly, the suppressing ability was confirmed by isolating the corresponding cDNA-carrying plasmids and reintroducing them back into the parental RE294 strain. An equal amount of transformants was spread on both repressing (*PR*-off) and inducing (*PR*-on) PMG plates supplemented with 300 mg/L of G418. The plasmid pYZ1N was used as a negative control. The suppressing plasmid cDNA inserts were then sequenced, and their putative gene functions were identified using Basic Local Alignment Search Tool (BLAST) homology searches of *S*. *pombe* genome databases.

### Mammalian cell assays

The effect of *hhp2* on *PR*-induced cell death was measured by staining HeLa cells with Trypan Blue, a dye that was used to specifically detect metabolically inert cells 24 hrs post-gene expression. The percentage of blue cells, dead cells, was determined under the microscope. The final counting results were calibrated by excluding the background and cytotoxicity caused by Hhp2. Two additional assays were used to measure HIV-1 PR-induced apoptosis in mammalian cells. The first method was Annexin V-PE staining using the BD Pharmingen PE Annexin V Apoptosis Detection Kit I (Cat. No. 559763, BD Biosciences, San Jose, CA) [22, 64]. Briefly, 24 hours after *PR* gene expression, aliquots of the transfected cells were collected and subjected to Annexin V staining and flow cytometric analysis. The staining was performed according to manufacturer’s protocol. Annexin V-PE fluorescent signals were detected using a Becton-Dickenson flow cytometer and analyzed with Cell Quest software. The percentage of apoptotic cells was calculated by excluding the background and cytotoxicity caused by Hhp2. The second method to measure HIV-1 PR-induced apoptosis was measuring the possible cleavages of pro-apoptotic proteins such as caspase-3 and caspase-8 [42, 43]. Forty-eight hours after *PR* gene expression, the transfected cells were collected and the levels of caspase-3 and caspase-8 were measured by western blot analysis [25]. The antibodies used were caspase-8 (1C12) mouse mAb (Cat#9746, Cell Signaling Technology), cleaved caspase-3 (Asp175) (5A1) rabbit mAb (Cat#9664, Cell Signaling Technology), and anti-β-actin mouse mAb (A2228, Sigma-Aldrich), which was used as a control. HIV-1 protease antiserum (Cat number: 4105) was obtained from NIH AIDS Reagent Program. A semi-quantitative RT-PCR assay to detect *hhp2* mRNA expressed in HeLa cells was performed using the SuperScript^™^ One-Step RT-PCR System (Cat. No. 10928–034, Invitrogen). The primers used were: 5'- ACG GTT GTT GAC ATT AAG -3' and 5'- AGG AGC TGG TTC TTC ATC -3'.

## Supporting Information

S1 Fig Construction of HIV-1 *PR*-carrying fission yeast strain RE294. The schematic diagram shows the process of creating a fission yeast strain that contains in its chromosome an integrated copy of the HIV-1 *PR* gene at the *nmt1* gene locus. Specifically, the wild type HIV-1 *PR* gene was amplified by PCR from a plasmid containing the entire genome of the HIV-1 NL_4-3_ laboratory strain. The amplified gene product was ligated between the fission yeast *nmt1* gene promoter and the kanamycin-resistant gene (kan^r^) marker on a plasmid. This plasmid construct was integrated at the *nmt1* locus by homologous gene recombination in the SP223 fission yeast strain. The new fission yeast strain was then named RE294.
(TIF)
