# Supplementary figures and images for: HIV-1 Protease in the Fission Yeast Schizosaccharomyces pombe
Source: PLoS One. 2016 Mar 16;11(3):e0151286. doi: 10.1371/journal.pone.0151286 (PMC4794156; doi:10.1371/journal.pone.0151286)

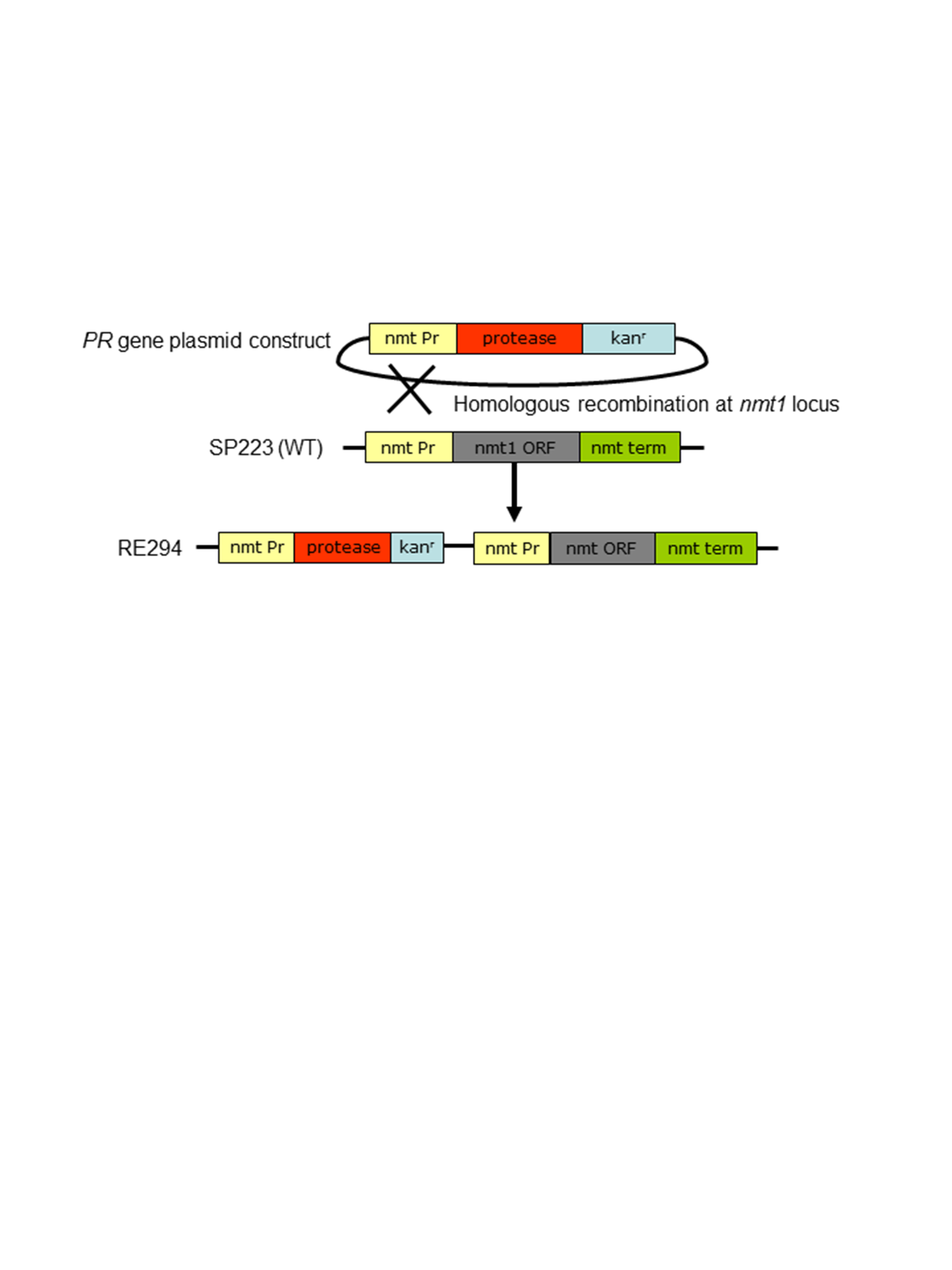

Supplement: S1 Fig — The schematic diagram shows the process of creating a fission yeast strain that contains in its chromosome an integrated copy of the HIV-1 PR gene at the nmt1 gene locus. Specifically, the wild type HIV-1 PR gene was amplified by PCR from a plasmid containing the entire genome of the HIV-1 NL4-3 laboratory strain. The amplified gene product was ligated between the fission yeast nmt1 gene promoter and the kanamycin-resistant gene (kanr) marker on a plasmid. This plasmid construct was integrated at the nmt1 locus by homologous gene recombination in the SP223 fission yeast strain. The new fission yeast strain was then named RE294. (TIF) [file pone.0151286.s001.tif]
